# Overexpression of PEAK1 contributes to epithelial–mesenchymal transition and tumor metastasis in lung cancer through modulating ERK1/2 and JAK2 signaling

**DOI:** 10.1038/s41419-018-0817-1

**Published:** 2018-07-23

**Authors:** Chenbo Ding, Wendong Tang, Xiaobo Fan, Xiyong Wang, Hairu Wu, Hongbo Xu, Wei Xu, Wei Gao, Guoqiu Wu

**Affiliations:** 1 Medical School of Southeast University, Nanjing, 210009 China; 2 Center of Clinical Laboratory Medicine, Zhongda Hospital, Southeast University, Nanjing, 210009 China

## Abstract

Pseudopodium-enriched atypical kinase 1 (PEAK1), a novel non-receptor tyrosine kinase, has been demonstrated to act as an oncogenic regulator in breast and pancreatic cancers. However, the role of PEAK1 in the progression and metastasis of lung cancer is still unknown. Here, we observed that ectopic PEAK1 expression promoted lung cancer cell migration and invasion, while PEAK1 knockout resulted in suppressed cell migration and invasion. Interestingly, cell proliferation did not significantly increase or decrease in either the PEAK1 overexpression or knockout groups compared with the corresponding control cells. In addition, PEAK1 overexpression could induce epithelial-to-mesenchymal transition (EMT) and the expression of matrix metalloproteinase-2 (MMP2) and MMP9 both in vitro and in vivo, whereas PEAK1 knockout had the opposite effects. Then, we had confirmed that PEAK1 was significantly upregulated in lung cancer tissues, and correlated with a higher tumor node metastasis stage. Moreover, PEAK1 upregulation markedly enhanced the activation of extracellular signal-regulated kinase-1/2 (ERK1/2) and Janus kinase-2 (JAK2) signaling in lung cancer cells. Further work demonstrated that the combination of PD98059 with AZD1480 could reverse the effects of PEAK1-induced EMT, cell migration and invasion. Our findings highlight a newer mechanism for PEAK1 in regulating EMT and metastasis in lung cancer, which might serve as a therapeutic target for lung cancer patients.

## Introduction

Lung cancer is the most frequently diagnosed malignance and the main cause of cancer-related death in the USA, China and other countries^1,2^. Approximately 85% of lung cancer patients are diagnosed with non-small cell lung cancer (NSCLC)^3^, and more than 80% of NSCLC cases are diagnosed at an advanced stage with activating epidermal growth factor receptor (EGFR) mutations^4^. Currently, cisplatin plus gemcitabine is a standard chemotherapy regimen for the first-line treatment of advanced NSCLC^5^. However, there is a serious problem of an increasing number of patients developing therapeutic resistance due to long-term chemotherapy and the occurrence of metastasis. It has been widely identified that epithelial–mesenchymal transition-inducing transcription factors (EMT-TFs), matrix metalloproteinases (MMPs) and signaling cascades are directly or indirectly involved in cancer cell metastasis^6,7^. EMT allows NSCLC cells to acquire invasive properties and to develop metastatic growth characteristics, and therapeutic resistance^6^. Thus, a better understanding of the molecular mechanisms underlying EMT and EMT-related traits in NSCLC is needed to improve early diagnosis and develop novel therapeutic strategies for NSCLC.

Protein tyrosine kinases (PTKs) are a class of kinases that catalyze the phosphorylation of tyrosine residues of various substrate proteins, and the development of tyrosine kinase inhibitors (TKIs) has transformed cancer therapy approaches^8^. PEAK1 (pseudopodium-enriched atypical kinase 1, also known as Sugen kinase 269 or Sgk269), belonging to new kinase family three (NKF3), is a catalytically active non-receptor TK and ubiquitously expresses in multiple tissues and organs^9^. PEAK1 is reported to contain several tyrosines within potential binding motifs and substrate residues for Src, extracellular signal-regulated kinase (ERK), Crk, and Shc proteins, which play important roles in regulating cell proliferation, migration, and apoptosis^9,10^. Recent works have suggested that PEAK1 plays a positive role in human pancreatic ductal adenocarcinoma (PDAC) growth, metastasis and therapy resistance^11–13^. In addition, PEAK1 regulates transforming growth factor beta (TGF-β) response and potentiates TGFβ-induced EMT, cell migration and metastasis in breast cancer^14,15^. However, the role of PEAK1 in the growth and metastasis of lung cancer has not been previously investigated.

In this study, we show that PEAK1 overexpression promotes lung cancer metastasis, EMT and EMT-related traits through regulating ERK1/2 and Janus kinase-2 (JAK2) signaling. The expression of PEAK1 was obviously higher in lung cancer tissues than in normal tissues, and positively associated with lymph node (LN) metastasis in clinical specimens. Finally, we also demonstrate that inhibitors of the ERK1/2 and JAK2 pathways could reverse PEAK1-induced EMT effects. These results provide new insights into the regulatory mechanism of EMT in lung cancer, as well as a novel therapeutic target.

## Results

### PEAK1 promotes NSCLC cell migration and invasion in vitro

**Fig. 1:**
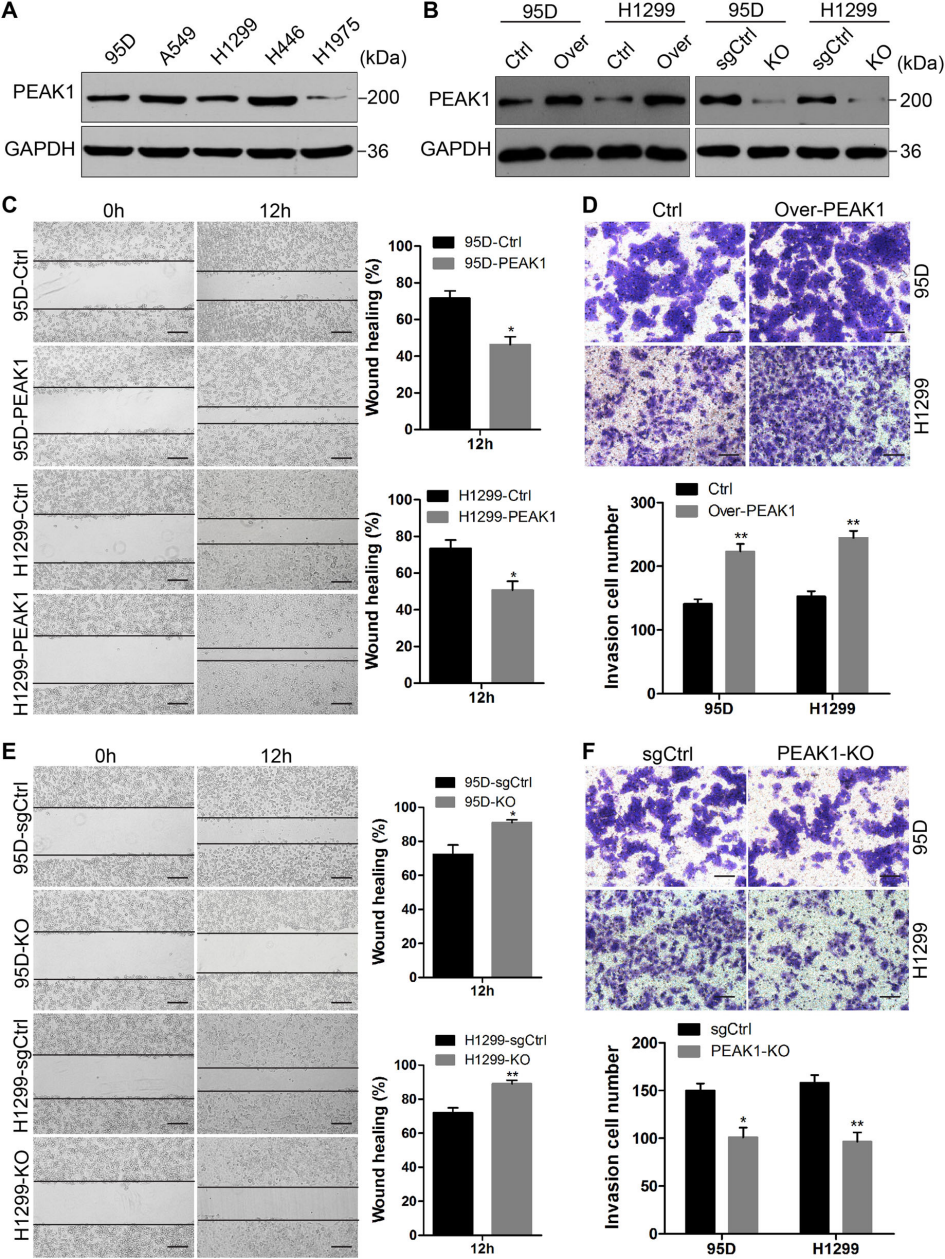
PEAK1 enhances NSCLC cell migration and invasion in vitro. **a** Western blot analysis of PEAK1 protein expression in five human lung cancer cell lines. **b** After cells were infected with LV-PEAK1, LV-PEAK1-sgRNA and corresponding control vectors, the expression of PEAK1 protein was detected by western blot analysis. **c** The migration of 95D-Ctrl, 95D-PEAK1, H1299-Ctrl, and H1299-PEAK1 cells was detected by scratch wound-healing assay. **d** Invasion assay of 95D-Ctrl, 95D-PEAK1, H1299-Ctrl, and H1299-PEAK1 cells. **e**, **f** Migration and invasion assay of 95D-sgCtrl, 95D-KO, H1299-sgCtrl, and H1299-KO cells (two clones, **c** and **d**). Scale bar, 100 μm. *P < 0.05; **P < 0.01

The level of PEAK1 protein in five human lung cancer cell lines (H1975, H1299, H446, 95D, and A549) was detected using western blot analysis. The lowest PEAK1 level was found in H1975 cells, while the highest level was found in H446 cells (Fig. 1a). Considering the experimental results, we chose to upregulate and silence PEAK1 expression in 95D and H1299 cell lines, which had moderate PEAK1 expression (Fig. 1b). To determine the effects of PEAK1 on lung tumor cell proliferation, migration and invasion, we performed Cell Counting Kit-8 (CCK-8), scratch wound-healing and transwell invasion assays, respectively. As results, overexpressing of PEAK1 enhanced 95D and H1299 cell migration and invasion (Fig. 1c, d), whereas PEAK1 knockout reduced lung cancer cell migration and invasion (Fig. 1e, f). However, neither upregulated nor silenced PEAK1 expression significantly affected cell proliferation (Fig. S1). These results suggest that PEAK1 promotes cell migration and invasion in NSCLC.

### PEAK1 induces EMT in NSCLC cells

**Fig. 2:**
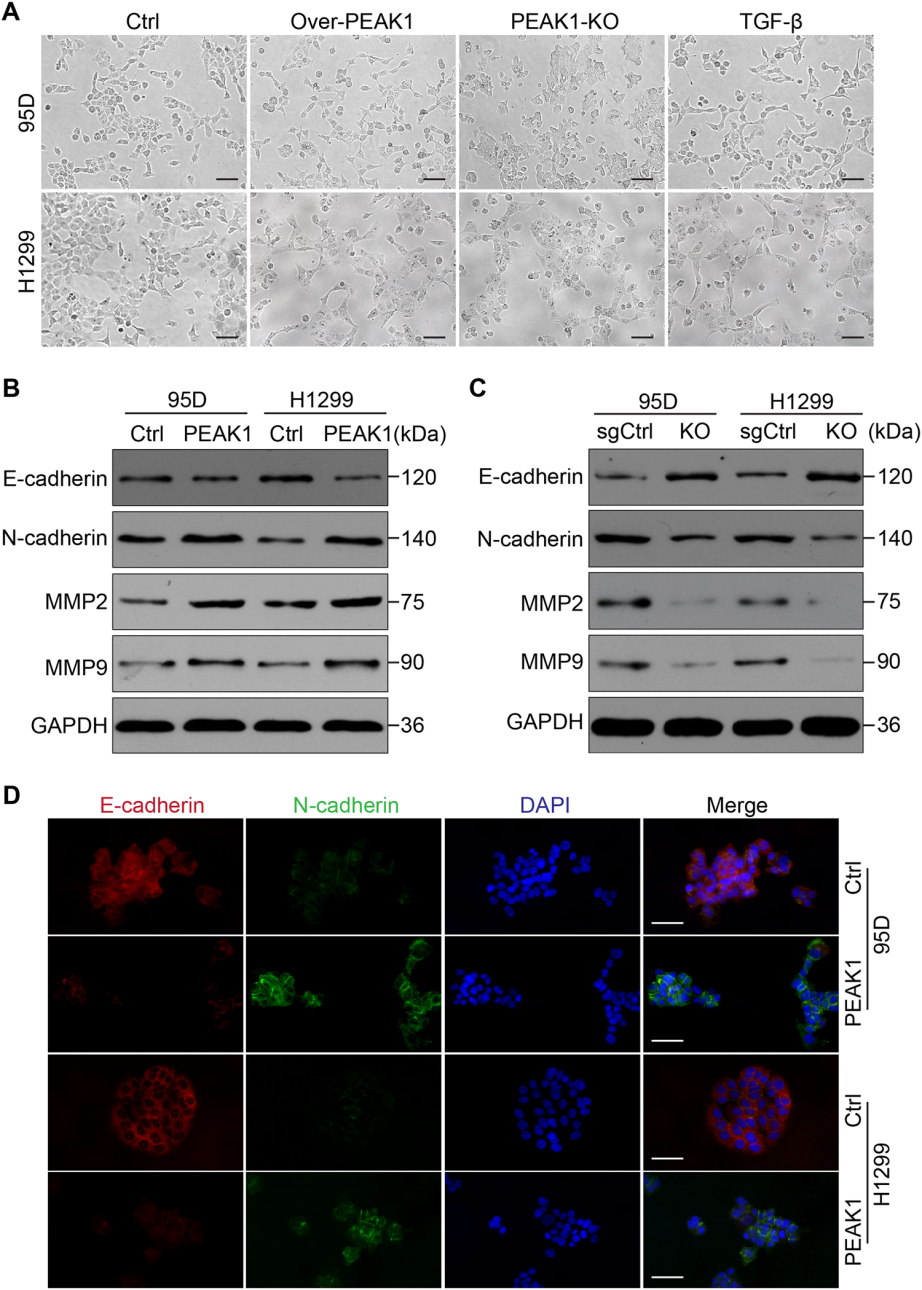
PEAK1 expression promotes EMT in NSCLC cells. **a** Morphological changes induced by PEAK1 expression in 95D and H1299 cells compared with TGF-β (10 ng/ml) treatment for 24 h. Scale bar, 100 μm. **b** Western blot showing decreased E-cadherin expression and increased N-cadherin expression, MMP2 and MMP9 expression in 95D-PEAK1 and H1299-PEAK1 cells compared with the corresponding control cells. **c** Western blot assay of E-cadherin, N-cadherin, MMP2 and MMP9 expression in 95D-sgCtrl, 95D-KO, H1299-sgCtrl and H1299-KO cells. **d** Immunofluorescence assay for E-cadherin and N-cadherin expression in 95D-Ctrl, 95D-PEAK1, H1299-Ctrl and H1299-PEAK1 cells. Scale bar, 150 μm

EMT is a development program that initiates metastatic dissemination of cancer cells from the primary tumor, and promotes mesenchymal phenotypes with increased migratory and invasive capabilities^16^. It is a complex process that can inhibit cell–cell connections and apicobasolateral polarity in epithelial cells, with decreased expression of epithelial markers (such as E-cadherin and β-catenin) and increased expression of mesenchymal markers (such as vimentin and N-cadherin)^17,18^. To explore the effect of PEAK1 expression on the EMT program, we compared the morphology of the cell models described above with that resulting from TGF-β-induced EMT. The results demonstrated that PEAK1 overexpression promoted a fibroblast-like mesenchymal phenotype in 95D and H1299 cells with a loss of cell–cell junctions, similar to the TGF-β-induced morphology. Conversely, PEAK1 knockout in 95D and H1299 cells resulted in a closer cell–cell adhesion and an epithelial cell-like morphology (Fig. 2a).

**Fig. 3:**
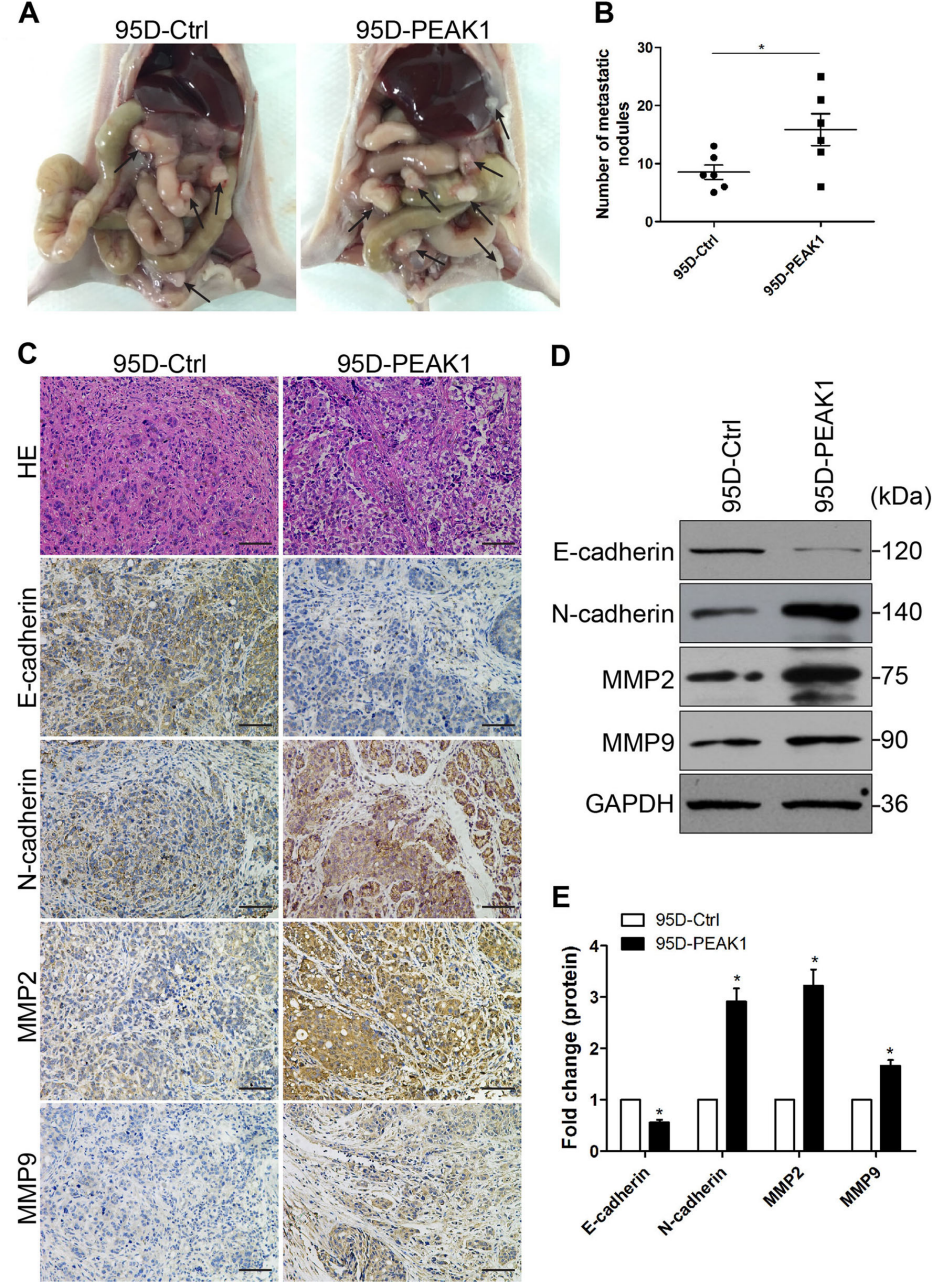
PEAK1 induced NSCLC cell metastasis and EMT in a mouse model. **a** Representative images of peritoneal dissemination in the 95D-Ctrl and 95D-PEAK1 groups. **b** Peritoneal dissemination metastatic nodules were counted and analyzed by Student’s *t*-test. **c** Tumor cells showed marked pleomorphism and multinucleation by H&E staining with PEAK1 upregulation, and peritoneal dissemination tumor nodules showed clearly increased N-cadherin, MMP2 and MMP9 expression and decreased E-cadherin expression upon PEAK1 upregulation compared with the controls as analyzed by immunohistochemistry. Scale bar, 100 μm. **d** Western blot analysis of E-cadherin, N-cadherin, MMP2 and MMP9 in peritoneal metastatic nodules. **e** The levels of E-cadherin, N-cadherin, MMP2 and MMP9 were calculated. *P < 0.05

It has shown that TGF-β/PEAK1 signaling is participated in breast cancer cells metastasis and EMT^14,15^. However, it is not clear whether the TGF-β/PEAK1 pathway is involved in the EMT process in lung cancer. Hence, we firstly explored whether TGF-β-induced EMT can affect the expression of PEAK1 levels in NSCLC cells. Unfortunately, TGF-β did not markedly affect the levels of PEAK1 in the EMT process (Fig. 2S). Coincident with these results, PEAK1 overexpression was not obviously upregulated the expression of TGF-β1/2/3 in NSCLC cells (Fig. 3S). Then, we detected the expression of the EMT-related markers E-cadherin and N-cadherin in cells with induced PEAK1 expression. The expression levels of E-cadherin and N-cadherin were significantly decreased and increased, respectively, in 95D and H1299 cells overexpressing PEAK1 (Fig. 2b and S4A). In contrast, PEAK1 knockout markedly decreased the expression of N-cadherin, but significantly increased the expression of E-cadherin in 95D and H1299 cells (Fig. 2c and S4B). Similar results were obtained by immunofluorescence (Fig. 2d). Furthermore, PEAK1 overexpression could induce the expression of MMP2 and MMP9, whereas PEAK1 knockout had the opposite effects (Fig. 2b, c). These results suggest that PEAK1 contributes to the acquisition of EMT characteristics and the EMT-derived invasive phenotype in NSCLC cells independent from the TGF-β level.

### PEAK1 facilitates NSCLC metastasis and EMT in vivo

According to our cell-based results, we speculated that PEAK1 overexpression could facilitate metastasis in animals. To examine whether PEAK1 enhances metastasis in vivo, peritoneal dissemination models of metastasis were established. After 40 days, the mice were anaesthetized, and metastatic nodules in the peritoneal cavity were collected; haematoxylin and eosin (H&E)-staining was performed to evaluate the tissue morphology. More metastatic nodules were found in the peritoneal cavity mice injected with PEAK1-overexpressing cells than in the controls (Fig. 3a, b). Metastatic colonies were collected from the peritoneal cavity in each group and evaluated for the expression of EMT-markers and MMPs. We found stronger N-cadherin, MMP2 and MMP9 staining, but weaker E-cadherin staining in metastatic colonies from the PEAK1-overexpressing group than in those from the controls (Fig. 3c). Similar results were obtained by western blot analysis (Fig. 3d, e). On the contrary, PEAK1 knockout had the opposite effects on tumor metastasis and EMT in vivo (data not shown). These in vivo results combined with the in vitro data reveal that PEAK1 promotes tumor metastasis and EMT.

### PEAK1 is upregulated in human lung cancer tissues and associated with metastasis

**Fig. 4:**
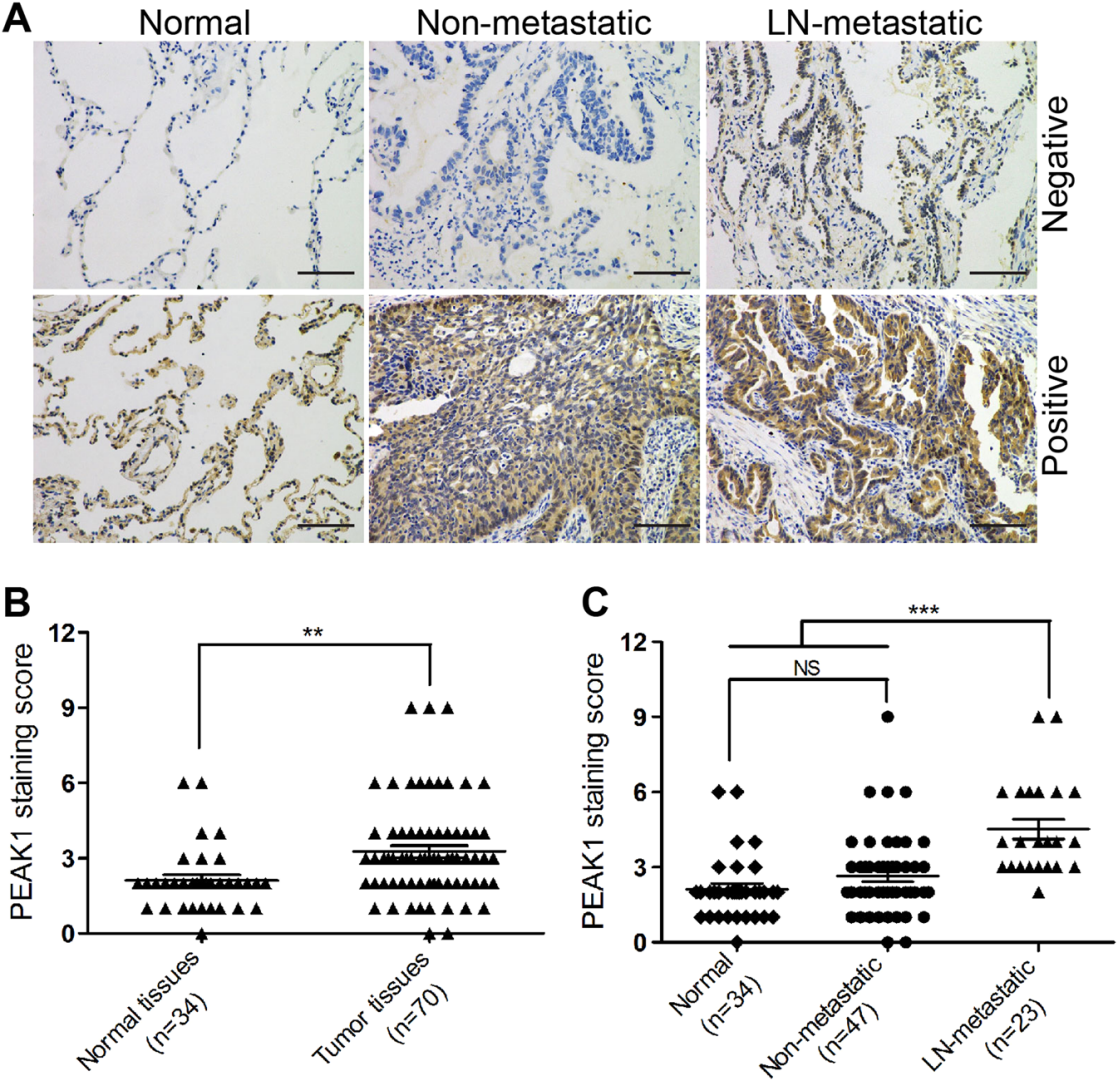
PEAK1 is markedly upregulated in metastatic lung cancer tissues. **a** Immunohistochemistry analysis of PEAK1 expression in lung cancer tissue and adjacent normal tissue. Scale bar, 100 μm. **b** Immunohistochemical staining of normal and tumor tissues was evaluated by staining scores. **c** Immunohistochemical staining of normal, non-metastatic tissues and LN-metastatic tissues was evaluated by staining scores. NS no statistical significance; **P *<* 0.01; ***P < 0.001

To explore whether PEAK1 is overexpressed in lung cancer tissues, we analyzed the expression of PEAK1 protein in 70 lung cancer and 34 adjacent non-tumor tissues. Immunohistochemical staining showed that PEAK1 was localized to both the cytoplasm and the membrane (Fig. 4a). According to the established principles for evaluating immunostaining, the rate of PEAK1-positive staining was significantly higher in the human lung cancer tissues than the adjacent non-tumor tissues (Fig. 4a, b and Table S1). Further analysis revealed that the staining score of PEAK1 was obviously higher in primary sites of lung cancer with LN metastasis than in those of non-metastatic lung cancer and in normal samples (Fig. 4c). Although the expression of PEAK1 in non-metastatic lung cancer tissues was higher than that in adjacent non-tumor tissues, there were no significant differences (Fig. 4c). These data indicate that PEAK1 is overexpressed in human lung cancer tissues, especially in metastatic lung cancer.

Next, to investigate whether PEAK1 expression is associated with the progression of NSCLC, we analyzed the correlations of PEAK1 expression with the clinicopathological characteristics of the lung cancer patients based on the immunohistochemical staining data (except for two cases of small cell lung cancer). As shown in Table 1, PEAK1 overexpression did not correlate with age, tumor size, histology, tumor differentiation or TNM stage (*P* > 0.05), but was significantly associated with gender and LN metastasis (*P* < 0.05). Collectively, these observations indicate that the upregulation of PEAK1 is positively correlated with metastasis in NSCLC.

**Table 1:** Correlations between PEAK1 expression and clinicopathological characteristics in 68 NSCLC patients

Characteristics	Total	PEAK1		*P*-value
		Negative	Positive	
Gender				0.016
Male	45	12	33	
Female	23	13	10	
Age				0.259
≤60	19	9	10	
>60	49	16	33	
Tumor size				0.618
<4 cm	55	21	34	
≥4 cm	13	4	9	
Histology				0.455
Adenocarcinomas	48	19	29	
Squamous cell carcinomas	20	6	14	
Tumor differentiation				0.087
Well-moderate	59	24	35	
Poor	9	1	8	
Lymph node metastasis				<0.001
Absent	45	24	21	
Present	23	1	22	
TNM stage				0.581
I–II	49	19	30	
III–IV	19	6	13	

### PEAK1 activates the ERK1/2 and JAK2 signaling pathways in NSCLC cells

**Fig. 5:**
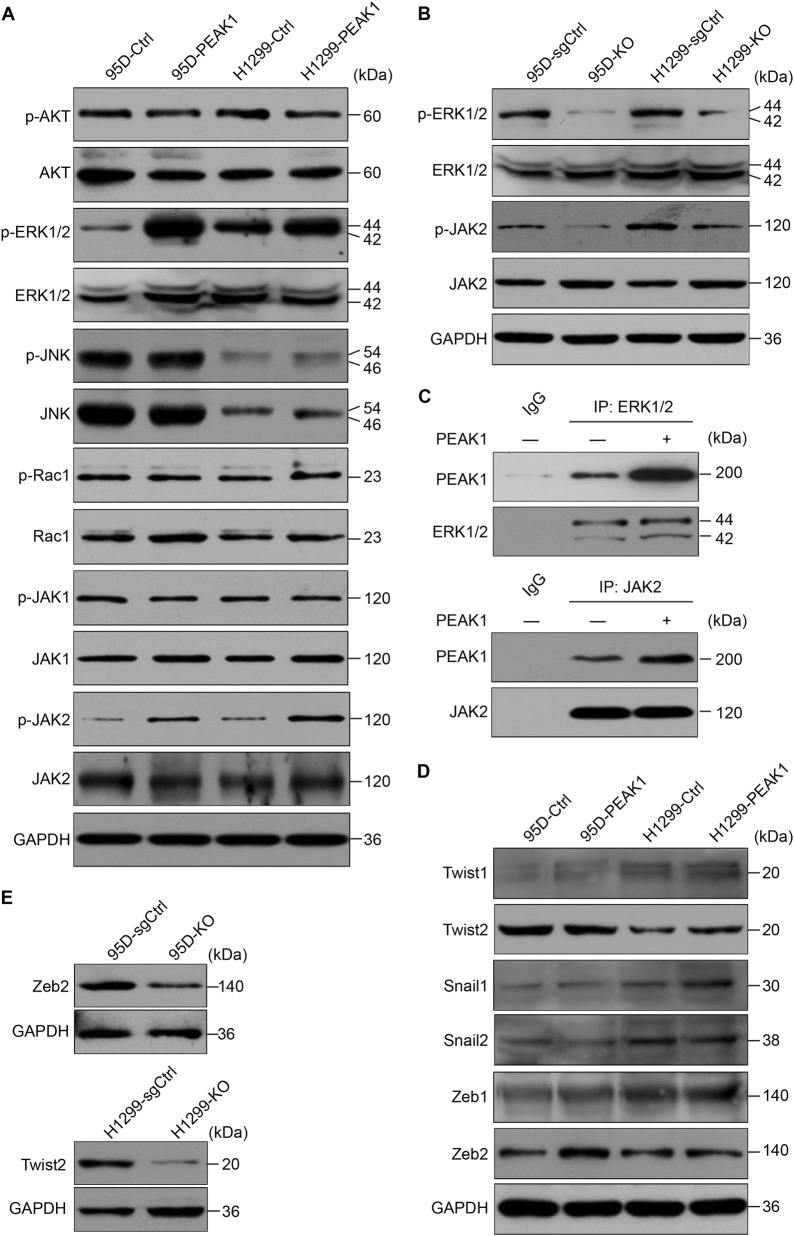
PEAK1 expression positively regulates ERK1/2 and JAK2 signaling, and induces the expression of Zeb2 or Twist2 in NSCLC cells. **a** PEAK1 overexpression activated the ERK1/2 and JAK2 signaling pathways, but did not affect the activation of AKT, JNK, Rac1 or JAK1. **b** PEAK1 knockout inhibited ERK1/2 and JAK2 signaling activation. **c** Control (−) or PEAK1-overexpressing (+) 95D cell lysates were detected by IP with nontarget IgG (negative control), anti-ERK1/2 or anti-JAK2 antibody, and blotting for PEAK1 associated proteins. IP immunoprecipitation. **d** PEAK1 overexpression induced Zeb2 or Twist2 expression in different NSCLC cells. **e** PEAK1 knockout suppressed Zeb2 or Twist2 expression in different NSCLC cells

The above results indicate that PEAK1 overexpression might play a critical role in NSCLC metastasis. However, the signals related to PEAK1 eliciting these biological effects in NSCLC remain unknown. Based on actin-targeting and kinase domains as well as the predicted binding or substrate residues, PEAK1 may mainly mediate p130Cas/Crk/Rac1 and mitogen-activated protein kinase (MAPK) signaling pathways^9,19^. In addition, its homolog Sgk223 has been reported to enhance the invasion of pancreatic ductal epithelial cells through JAK1/Stat3 signaling^20^. Therefore, we simultaneously examined the effects of PEAK1 on activation of the serine/threonine kinase (AKT), MAPKs, Rac1 and JAK1/2 signaling pathways in NSCLC cells. The phosphorylation of AKT, c-Jun N-terminal kinase (JNK), Rac1 and JAK1 was similar in the control and PEAK1-overexpressing cells (Fig. 5a and S5). However, ERK1/2 and JAK2 phosphorylation was significantly enhanced in PEAK1 overexpression cells compared with the control cells (Fig. 5a and S5). In contrast, the knockout of PEAK1 obviously reduced the activation of ERK1/2 and JAK2 in NSCLC cells (Fig. 5b and S5). Co-immunoprecipitation assays further indicated that PEAK1/ERK1/2 and PEAK1/JAK2 associations were markedly increased in PEAK1-overexpressing 95D cells (Fig. 5c). These data indicate that PEAK1 may enhance metastasis in NSCLC through directly targeting the ERK1/2 and JAK2 signaling pathways.

Several multipotent TFs, such as Twist1/2, Snail1/2 and Zeb1/2 play key roles in the regulation of EMT program and tumor metastasis^21^. To demonstrate whether PEAK1 induces ERK1/2 and JAK2 activation and then promotes TFs expression and EMT, we analyzed TFs expression at both the mRNA and protein levels in PEAK1-overexpressing and control cells. The mRNA and protein levels of Zeb2 and Twist2 were higher in PEAK1-overexpressing 95D and H1299 cells than in the corresponding control cells (Fig. 5d and S6), whereas PEAK1 knockout had the opposite effects (Fig. 5e and S6). These findings suggest that PEAK1 activates ERK1/2 and JAK2 signaling, which then promotes the expression of Zeb2 or Twist2 in NSCLC cells.

### Pharmacological inhibitors of ERK1/2 and JAK2 attenuate PEAK1-induced EMT, and cell migration and invasion

**Fig. 6:**
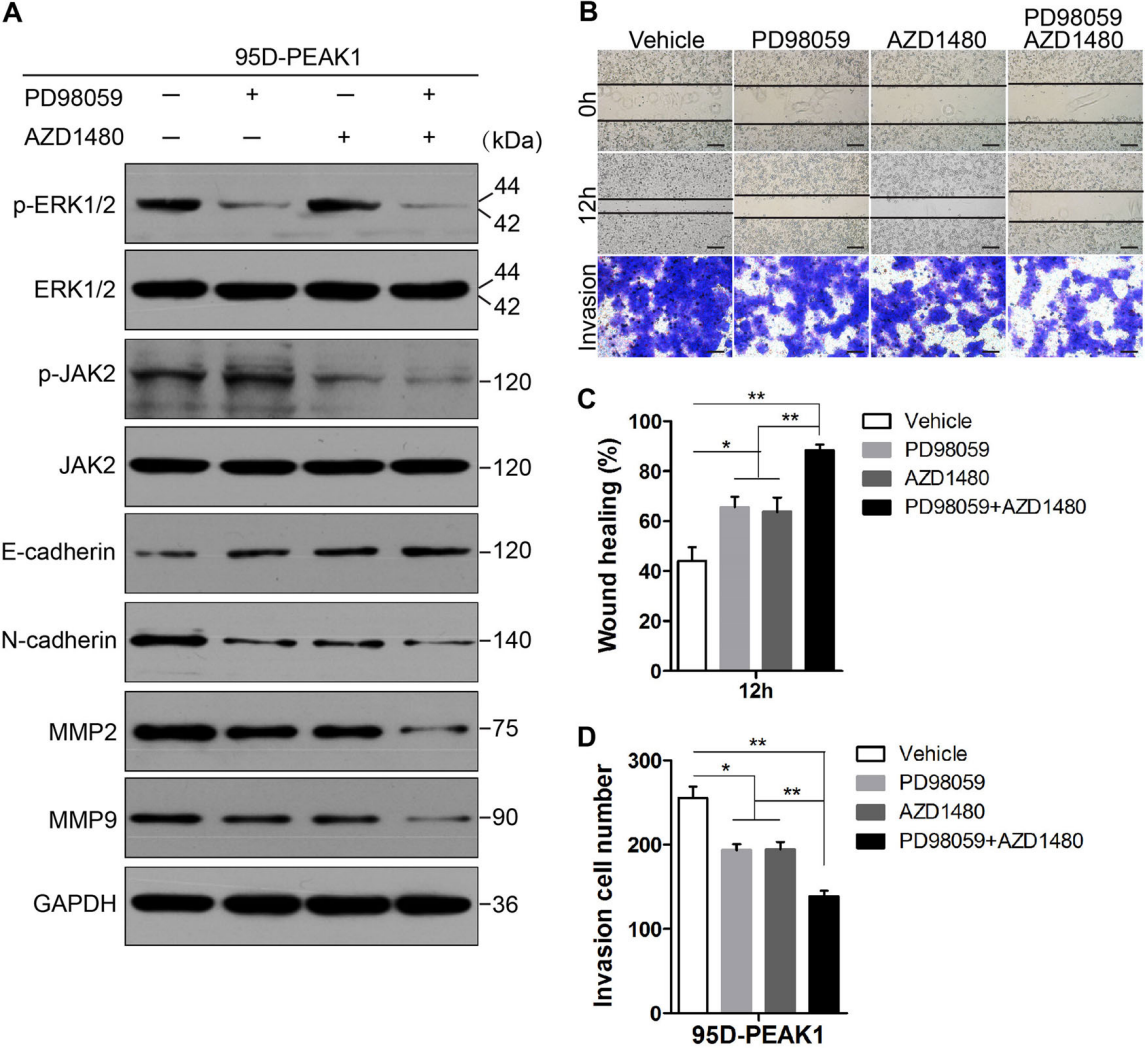
Inhibitors of ERK1/2 and JAK2 reduce PEAK1-enhanced EMT and cell migration and invasion. **a** 95D-PEAK1 cells were treated or not with 20 μM PD98059 and/or AZD1480 for 24 h after which E-cadherin, N-cadherin, MMP2, MMP9, p-ERK1/2, total ERK1/2, p-JAK2, and total JAK2 protein levels were analyzed by western blot. **b** 95D-PEAK1 cells were subjected to scratch wound-healing and invasion assays in the absence (vehicle) or presence of 20 μM PD98059 and/or AZD1480. Scale bar, 100 μm. **c**, **d** Migration and invasion of 95D-PEAK1 cells in the absence (vehicle) or presence of 20 μM PD98059 and/or AZD1480 were quantitatively analyzed. Columns are the average of three independent experiments ± SEM. *P < 0.05; **P < 0.01

Small molecule inhibitors of ERK1/2 and JAK2 signaling are actively being tested for cancer therapies^22,23^. To find potential treatment for NSCLC with higher PEAK1 expression, we tested whether PD98059, an effective mitogen-activated protein kinase kinase (MEK) inhibitor, or AZD1480, a specific JAK2 inhibitor, could block PEAK1-induced EMT, cell migration and invasion in NSCLC. In PEAK1-overexpressing 95D (95D-PEAK1) cells, 24 h of treatment with PD98059 or AZD1480 inhibited PEAK1-mediated signaling in a concentration-dependent manner (Fig. S7). In addition, when the concentration of PD98059 or AZD1480 was greater than 40 μM, cell proliferation was obviously decreased (Fig. S8). Considering the experimental data, we chose moderate PD98059 and/or AZD1480 concentrations (20 μM) to explore EMT and the migration and invasion of NSCLC cells in vitro. As shown in Fig. 6a, the expression of E-cadherin was partially regained, and the expression of N-cadherin, MMP2, and MMP9 was decreased upon the treatment of 95D-PEAK1 cells with PD98059 or AZD1480. Furthermore, the expression of N-cadherin, MMP2, and MMP9 was obviously downregulated; and the expression of E-cadherin was remarkably upregulated upon treatment with both PD98059 and AZD1480 compared with PD98059 or AZD1480 alone (Fig. 6a and S9). Scratch wound-healing and transwell assays showed attenuated migration and invasion in 95D-PEAK1 cells treated with PD98059 or AZD1480 alone compared to the control cells (Fig. 6b–d). When PD98059 was combined with AZD1480, cell migration and invasion were further inhibited (Fig. 6b–d). These data reveal that PEAK1-induced EMT, and cell migration and invasion are dependent on ERK1/2 and JAK2 signaling in NSCLC.

## Discussion

PEAK1 upregulation has been observed in various cancers, and PEAK1 may function as an oncogene involved in tumorigenesis and cancer progression^11–15^. In this study, we provide convincing evidence of the oncogenic role of PEAK1 in metastasis and EMT progression in NSCLC. Our results suggest that PEAK1 overexpression promotes the migration and invasion of NSCLC cells, and induces EMT. PEAK1 expression could facilitate the activation of ERK1/2 and JAK2, and then enhance the expression of downstream genes, including MMP2, MMP9, Zeb2, or Twist2. Meanwhile, ectopic expression of PEAK1 promoted tumor metastasis and EMT in vivo. However, compared with PEAK1 overexpression, PEAK1 knockout showed the opposite effects on signaling activation, genes expression and cancer characteristics. Moreover, the levels of PEAK1 protein were obviously higher in the clinical lung cancer samples than the adjacent normal tissues, and PEAK1 overexpression was obviously associated with LN metastasis. Collectively, our results suggest that PEAK1 is an oncogenic tyrosine kinase in NSCLC.

Tumor metastasis is an outcome influenced by multiple factors, such as EMT, the tumor microenvironment and angiogenesis^24–26^. Biologically, EMT is a progression with the conversion of epithelial cells to a mesenchymal phenotype and the enhancements in cell invasion and metastasis^16^. The loss of E-cadherin and an increase in N-cadherin/vimentin expression are hallmarks of the EMT process, and transcription factors including Twist1/2, Snai1/2 and Zeb1/2 are involved in the program^17,21^. MMPs degrade and modify the extracellular matrix (ECM), as well as cell–ECM and cell–cell contacts, thereby facilitating not only cell motility and invasion but also EMT^27^. It has been reported that PEAK1 drives cell migration and tumor metastasis in PDAC and breast cancer^11,15^. In addition, studies have demonstrated that PEAK1 is necessary for TGFβ-induced EMT in breast cancer^14,15^. However, the exact mechanism of PEAK1 action in tumor metastasis and EMT remains unclear. Of note, our results indicate that PEAK1 overexpression enhanced NSCLC cell migration and invasion. Meanwhile, PEAK1 overexpression promoted the transformation of polarized epithelial cells into fibroblast-like mesenchymal cells with a decrease in cell–cell adhesion and E-cadherin expression, and an increase in N-cadherin, MMP2, MMP9, and Zeb2 or Twist2 expression in different NSCLC cells, independent from the TGF-β level. We further found that PEAK1 significantly promoted tumor metastasis and EMT in a mouse model. Conversely, PEAK1 knockout had the opposite effects both in vitro and in vivo. These data suggest that PEAK1 expression is closely associated with invasion and metastasis through the modulation of EMT.

Next, we assessed PEAK1 protein expression patterns in lung cancer samples from patients. Indeed, PEAK1 expression was higher in human lung cancer tissues than adjacent non-tumor tissues. Furthermore, PEAK1 expression was obviously increased in primary sites of metastatic lung cancer compared with either non-metastatic lung cancer or normal samples. To clarify whether there might be a positive correlation between PEAK1 and tumor metastasis, we compared the results with the clinicopathological characteristics of the patients. We found that PEAK1 expression was significantly associated with LN metastasis. Interestingly, the expression of PEAK1 was positively associated with the female sex. Most of the clinical specimens in our study were from female patients, and the incidence of lung cancer in women has significantly increased over recent years; this increase is mainly attributed to active and passive smoking, kitchen fumes and air pollution. It has been widely recognized that human breast tumor cells collectively induce the assembly of new tumor blood vessels, and breach lung capillaries to become circulating tumor cells and metastasize to the lungs, among other organs^28^. In addition, PEAK1 overexpression has been found in breast cancer and is involved in the progression and metastasis of breast cancer^15,29^. One plausible explanation is that the expression and function of PEAK1 might be affected by disordered estrogen secretion in breast and lung cancers.

The ERK1/2 and JAK2 signaling pathways participate in the progression and metastasis of lung cancer, and are attractive therapeutic targets for NSCLC^30–32^. Additionally, ERK1/2 and JAK2 phosphorylation promotes EMT and treatment-resistance in lung cancer^33,34^. To investigate the possible pathways underlying the participation of PEAK1 in EMT and metastasis in lung cancer, we applied western blot analysis. ERK1/2 and JAK2 phosphorylation was markedly greater in PEAK1-overexpressing NSCLC cells than the corresponding control cells. By contrast, PEAK1 knockout obviously inhibited the activation of ERK1/2 and JAK2 signaling. Further studies have shown that PEAK1 can co-immunoprecipitate with ERK1/2 and JAK2 in 95D cells. In addition, PD98059 and/or AZD1480 can reduce the PEAK1-enhanced migration, invasion and EMT of NSCLC cells, suggesting that targeting the ERK1/2 and JAK2 pathways could potentially be used to treat PEAK1-overexpressing lung cancer in combination with standard chemotherapy. Our findings reveal that PEAK1 overexpression contributes to activation of the ERK1/2 and JAK2 pathways and promotes EMT in a lung cancer subset.

Our data show that PEAK1 is significantly increased in lung cancers, and that its overexpression is associated with tumor metastasis. Increased PEAK1 expression can also induce EMT and promote NSCLC metastasis through ERK1/2 and JAK2 signaling. It is important to determine in future studies whether the expression of PEAK1 protein has any prognostic value in lung cancer patients. In addition, it has been shown that cancer stem cells (CSCs) have the molecular characteristics of cells in EMT and drug-resistant lung cancer^35^. It would be interesting to examine whether the PEAK1 expression status in lung cancer can mediate the CSC phenotype and predict the response to chemotherapy. In summary, this study demonstrates that PEAK1 exhibits a strong oncogenic effect in the progression and metastasis of NSCLC by facilitating the EMT program. Targeting PEAK1-mediated molecular mechanisms might be an effective therapeutic strategy for EGFR-TKI-based treatments in certain lung cancer subsets.

## Materials and methods

### Cell culture

Human lung cancer cell lines H1975, H1299, H446, 95D, and A549 were obtained from the Cell Bank of Chinese Academy of Sciences (Shanghai, China). All cells were maintained in RPMI-1640 medium (Gibco/Life Technologies, USA) supplemented with 10% fetal bovine serum (FBS, Invitrogen, USA) and 1% penicillin–streptomycin at 37 °C in a humidified atmosphere with 5% CO_2_.

### Clinical tissue collection

In total, 70 paraffin-embedded lung cancer samples and 34 normal lung tissue cases were collected from lung cancer patients undergoing pulmonary surgery at the Department of Cardiothoracic Surgery and Pathology of Zhongda Hospital between December 2015 and December 2016 and examined to validate the candidate. None of these patients had received chemo-, radio- or immunotherapy prior to surgery. Informed consent was obtained from all patients before surgery, and our study was approved by the Ethical Committee of Zhongda Hospital according to the 1975 declaration.

### DNA/lentiviral constructs and cell transfection

To construct the HA-PEAK1 overexpression vector, the full-length human PEAK1 gene was amplified from the template PEAK1 cDNA clone (GenScript Biotechnology, China) and subcloned into the pLVX-IRES-Puro lentiviral vector (Clontech, USA). For transfection, H1299 and 95D cells were seeded at 70–80% confluence; 12 h later, the cells were transiently transfected with indicated vectors with Lipofectamine® 3000 (Invitrogen, USA) according to the manufacturer’s instructions. The Lenti-CAS9-sgRNA system for PEAK1 knockout was constructed by GeneChem (Shanghai, China). Cell lines with stable overexpression or knockout were generated as previously described^36^. In addition, H1299 and 95D cells were infected with the lentiviral vector at a multiplicity of infection of approximately 50 with 6 μg/ml polybrene. After 15 h, the H1299 and 95D cell culture medium was replaced with fresh medium; puromycin (5 μg/ml) selection was performed after 96 h of infection. The relevant empty lentiviral-vectors were used as negative controls. The expression of PEAK1 was detected by western blot analysis.

### Immunohistochemical assay

Paraformaldehyde-fixed paraffin sections were subjected to immunohistochemical assays and staining was scored as previously reported^37,38^.

### RT-PCR analysis

Total RNA was isolated with the TRIzol reagent (Invitrogen, USA). mRNA expression was analyzed with SYBR® Premix Ex Taq™ II (TaKaRa, Japan) according to the manufacturer’s instructions. The mix was preheated at 95 °C (45 s), and then amplified at 95 °C (10 s) and 60 °C (40 s) for 40 cycles. PEAK1 primers: forward 5′-GTTCACAGAGGCGAAAGGAG-3′; reverse 5′-CTGGTCGTCCTAGCCTTGC-3′. E-cadherin primers: forward 5′-GAACGCATTGCCACATACAC-3′; reverse 5′-GAATTCGGGCTTGTTGTCAT-3′. N-cadherin primers: forward 5′-TGCCAGTGTGACTCCAACG-3′; reverse 5′-GCAGGATGATGATGCAGAGC-3′. Twist1 primers: forward 5′-GGCCAGGTACATCGACTTCC-3′; reverse 5′-CATCCTCCAGACCGAGAAGG-3′. Twist2 primers: forward 5′-GGCCGCCAGGTACATAGACT-3′; reverse 5′-ACGGAGAAGGCGTAGCTGAG-3′. Snail1 primers: forward 5′-GGCTCCTTCGTCCTTCTCCT-3′; reverse 5′-CTGGAGATCCTTGGCCTCAG-3′. Snail2 primers: forward 5′-GACTACCGCTGCTCCATTCC-3′; reverse 5′-TGGTCCTTGGAGGAGGTGTC-3′. Zeb1 primers: forward 5′-ACCTGCCAACAGACCAGACA-3′; reverse 5′-TCCTGCTTCATCTGCCTGAG-3′. Zeb2 primers: forward 5′-ATGACCTGCCACCTGGAACT-3′; reverse 5′-TCTCGTGGCGGTACTTGATG-3′. TGF-β1 primers: forward 5′-CCGTGGGATACTGAGACACC-3′; reverse 5′-GGTCTCCCGGCAAAAGGTAG-3′. TGF-β2 primers: forward 5′-TTGTGCTCCAGACAGTCCCA-3′; reverse 5′-GCTCAATCCGTTGTTCAGGC-3′. TGF-β3 primers: forward 5′-GTGCCGTGAACTGGCTTCT-3′; reverse 5′-CTTGGCGATGGGGAGAAAGT-3′. GAPDH primers: forward 5′-GAAGGTGAAGGTCGGAGTC-3′; reverse 5′-GAAGATGGTGATGGGATTTC-3′.

### Western blot analysis and immunoprecipitation

Western blot assays were performed as previously described^37^. The primary antibodies used in our study were as follows: PEAK1 (Santa Cruz Biotechnology, USA); PEAK1/NKF3, E-cadherin, N-cadherin, MMP2, and MMP9 (Abcam, UK); p-AKT, total AKT, p-ERK1/2, total ERK1/2, p-JNK and total JNK (Invitrogen, USA); p-Rac1, total Rac1, p-JAK1, total JAK1, p-JAK2, total JAK2, Twist1, Twist2, Snail1, Snail2, Zeb1, and Zeb2 (OriGene, USA); and GAPDH (Immunology Consultants Laboratory, USA). Immunoprecipitation was performed exactly as previously described^39^.

### CCK-8 assay

H1299 and 95D cells were seeded in 96-well plates at 1 × 10^4^/well in triplicate and transiently transfected with p-PEAK1 plasmid or the relevant control plasmid. In addition, stable PEAK1 knockout H1299 and 95D cells were cultured as previously described. At the indicated time points, cells were subjected to CCK-8 assay kit (Dojindo, Kumamoto, Japan), which was performed as previously reported^40^.

### Scratch wound-healing assay

Transiently overexpressing or stable PEAK1 knockout H1299 and 95D cells were seeded in 6-well plates at 5 × 10^5^/well. Then, a single scratch was made across the centre of the cell monolayer using a micropipette tip, and the cells were washed with phosphate-buffered saline to remove debris. Images were captured under a microscope at 12 h post wounding and the migrated cells were counted. Three independent experiments were performed in triplicate.

### Invasion assay

The transwell system with 24-well polycarbonate membranes and 8 μm pores (Corning Costar, USA) was used to perform invasion assays as previously reported^38^. Dimethyl sulphoxide (vehicle), PD98059 and/or AZD1480 were added to both the top and bottom chambers of each well.

### Immunofluorescence

Cells were cultured on glass slides and immunofluorescence staining was performed as previously described^41^.

### Animal experiment

All animal studies were conducted according to protocols approved by the Ethical Committee of the Medical School of Southeast University, People’s Republic of China. Female athymic BALB/c nude mice (4–5 weeks-old) were purchased from the Yangzhou University Animal Center (Yangzhou, China). To produce peritoneal dissemination metastatic models, stable PEAK1 overexpressing and knock out cells and the corresponding control cells were injected into the abdominal cavity of mice. After 40 days, all the mice were sacrificed under anesthesia. The peritoneally disseminated metastatic nodules were collected and fixed in 10% formalin or frozen at −80 °C until being processed for protein extraction. To evaluate the tissue morphology and EMT-related traits, the samples were sectioned and subjected to H&E staining, immunohistochemistry staining and western blot analyses.

### Statistical analysis

Differences between the experimental and control groups were assessed by Student’s *t*-test. Pearson’s *χ*^2^-test was used for clinical correlative studies. A *P*-value of 0.05 or less was considered statistically significant. Statistical analyses were computed using SPSS 19.0 and GraphPad Prism 5.0. All values are represented as the mean ± SEM from at least three independent experiments.

## Electronic supplementary material

Figure S1

Figure S2

Figure S3

Figure S4

Figure S5

Figure S6

Figure S7

Figure S8

Figure S9

Table S1

Supplementary Figure and Table Legends
