# Supplementary figures and images for: Overexpression of PEAK1 contributes to epithelial–mesenchymal transition and tumor metastasis in lung cancer through modulating ERK1/2 and JAK2 signaling
Source: Cell Death Dis. 2018 Jul 23;9(8):802. doi: 10.1038/s41419-018-0817-1 (PMC6056550; doi:10.1038/s41419-018-0817-1)

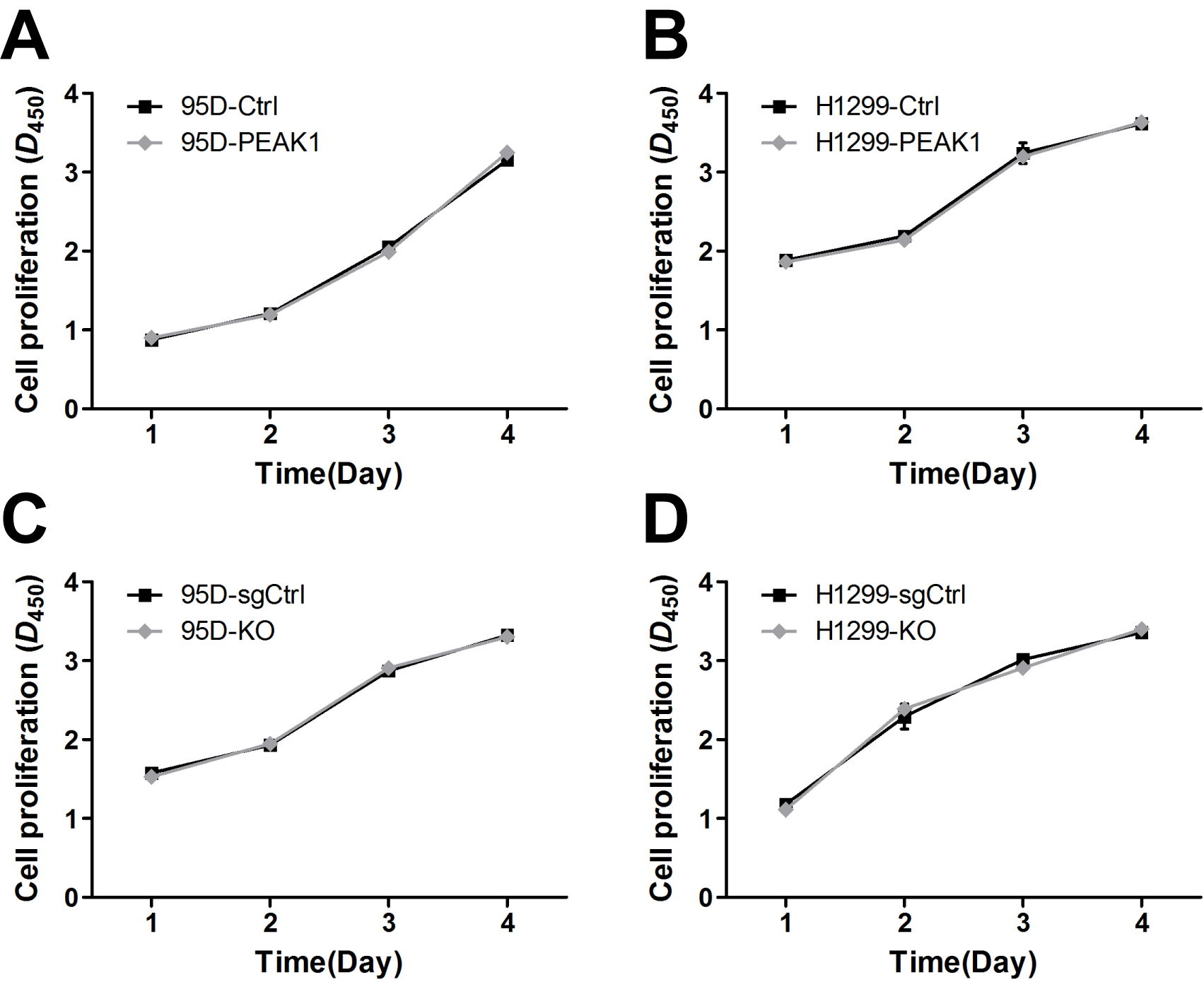

Supplement: Supplementary file 1 — Figure S1 [file 41419_2018_817_MOESM1_ESM.tif]

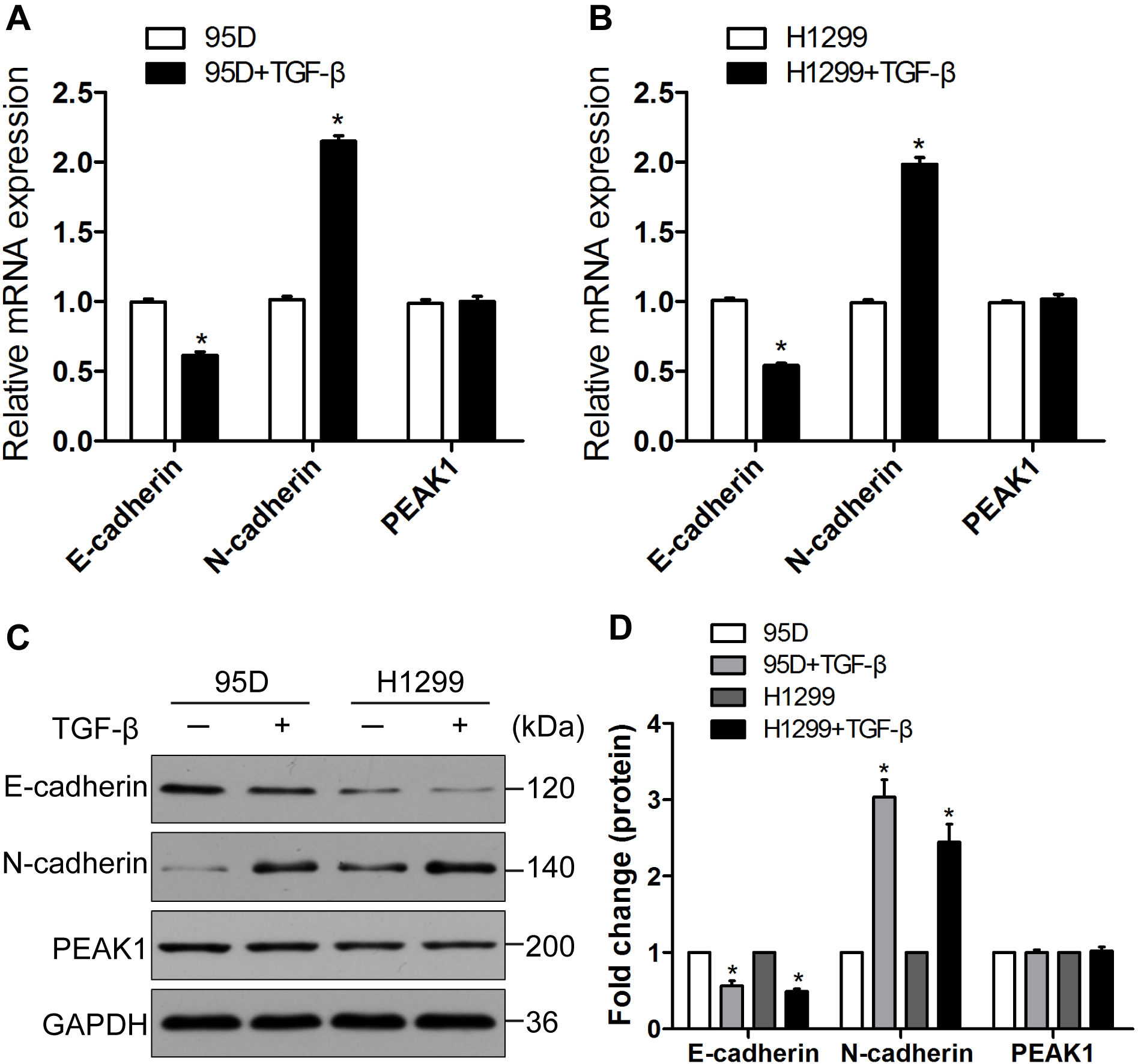

Supplement: Supplementary file 2 — Figure S2 [file 41419_2018_817_MOESM2_ESM.tif]

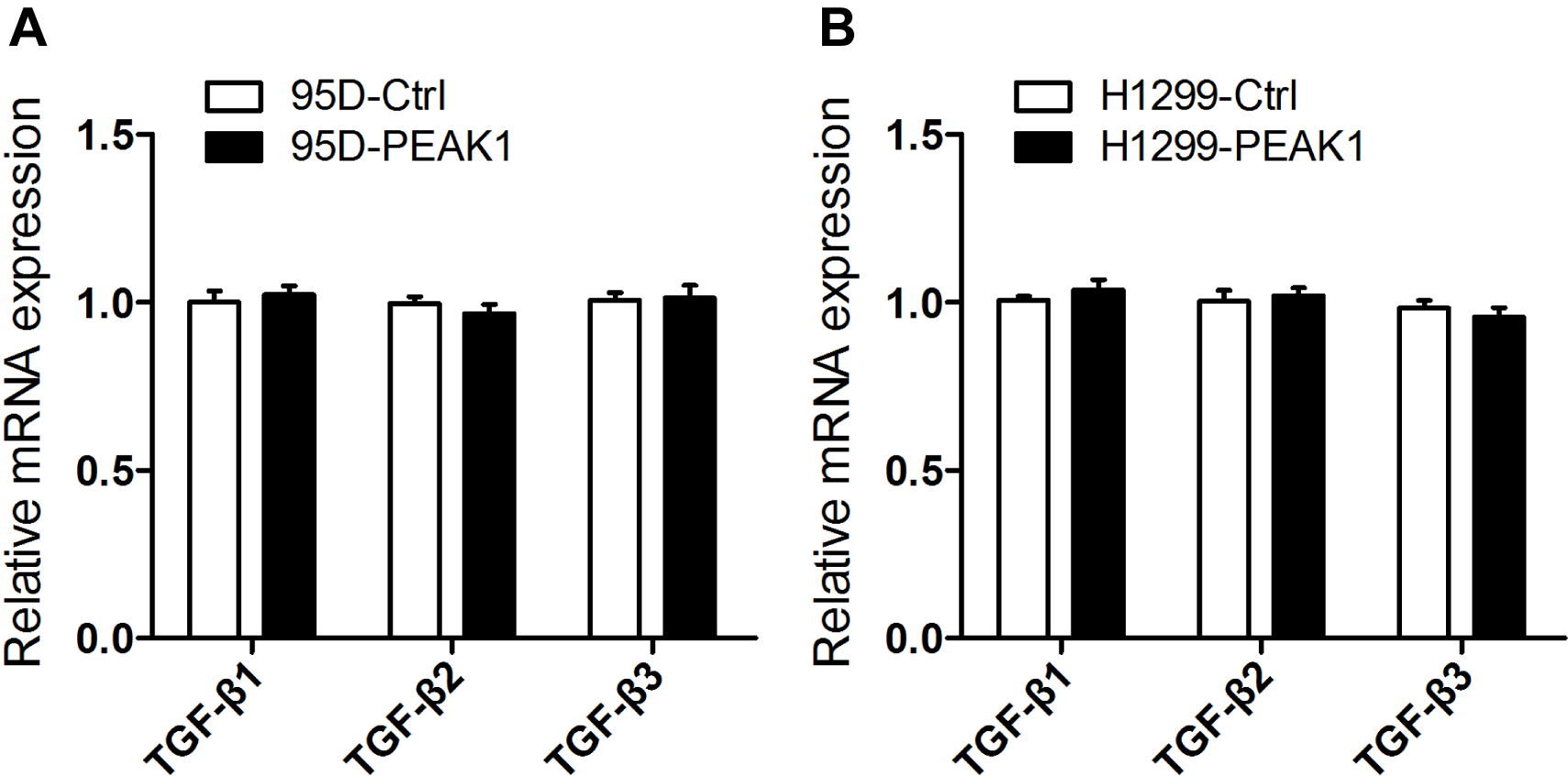

Supplement: Supplementary file 3 — Figure S3 [file 41419_2018_817_MOESM3_ESM.tif]

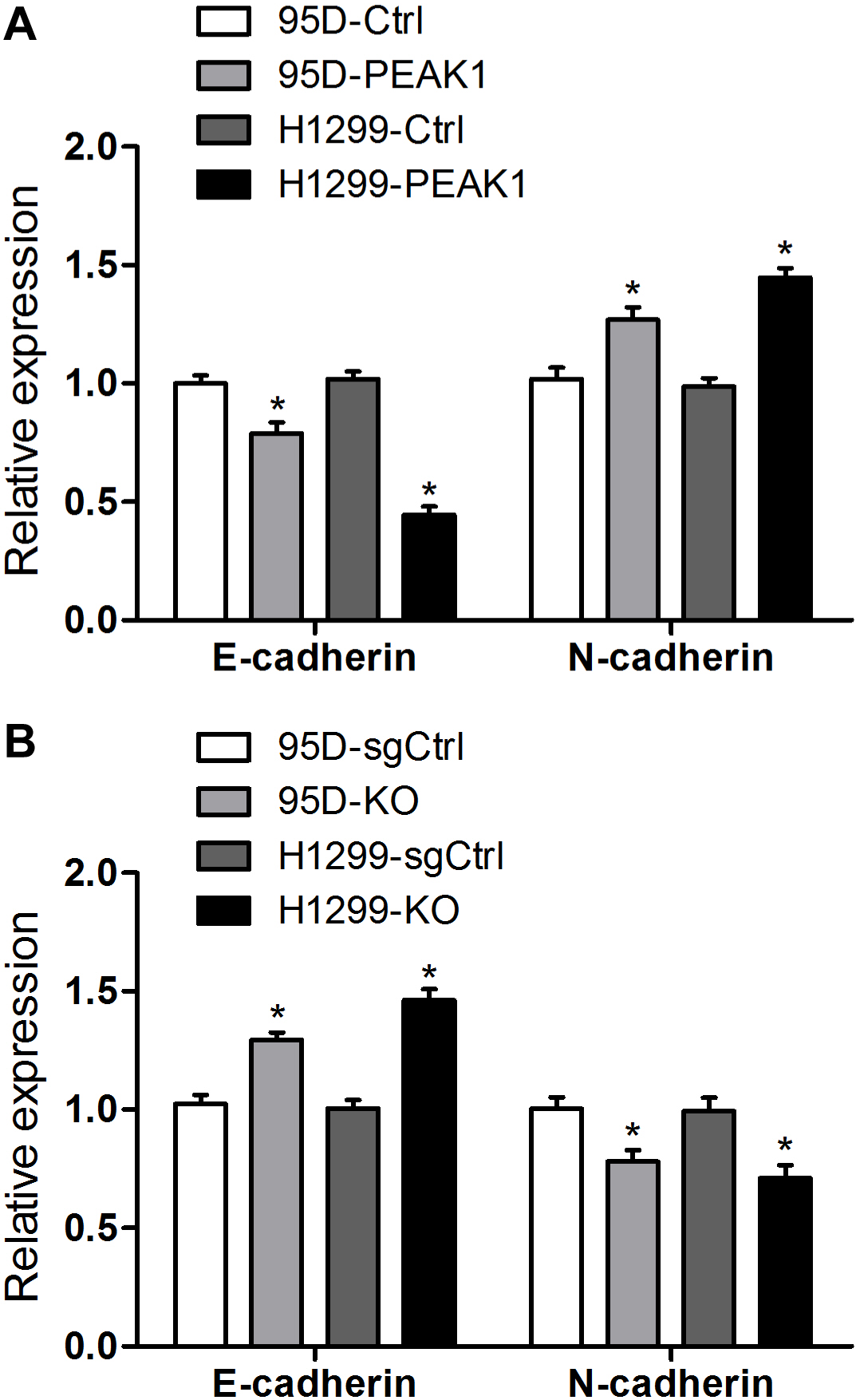

Supplement: Supplementary file 4 — Figure S4 [file 41419_2018_817_MOESM4_ESM.tif]

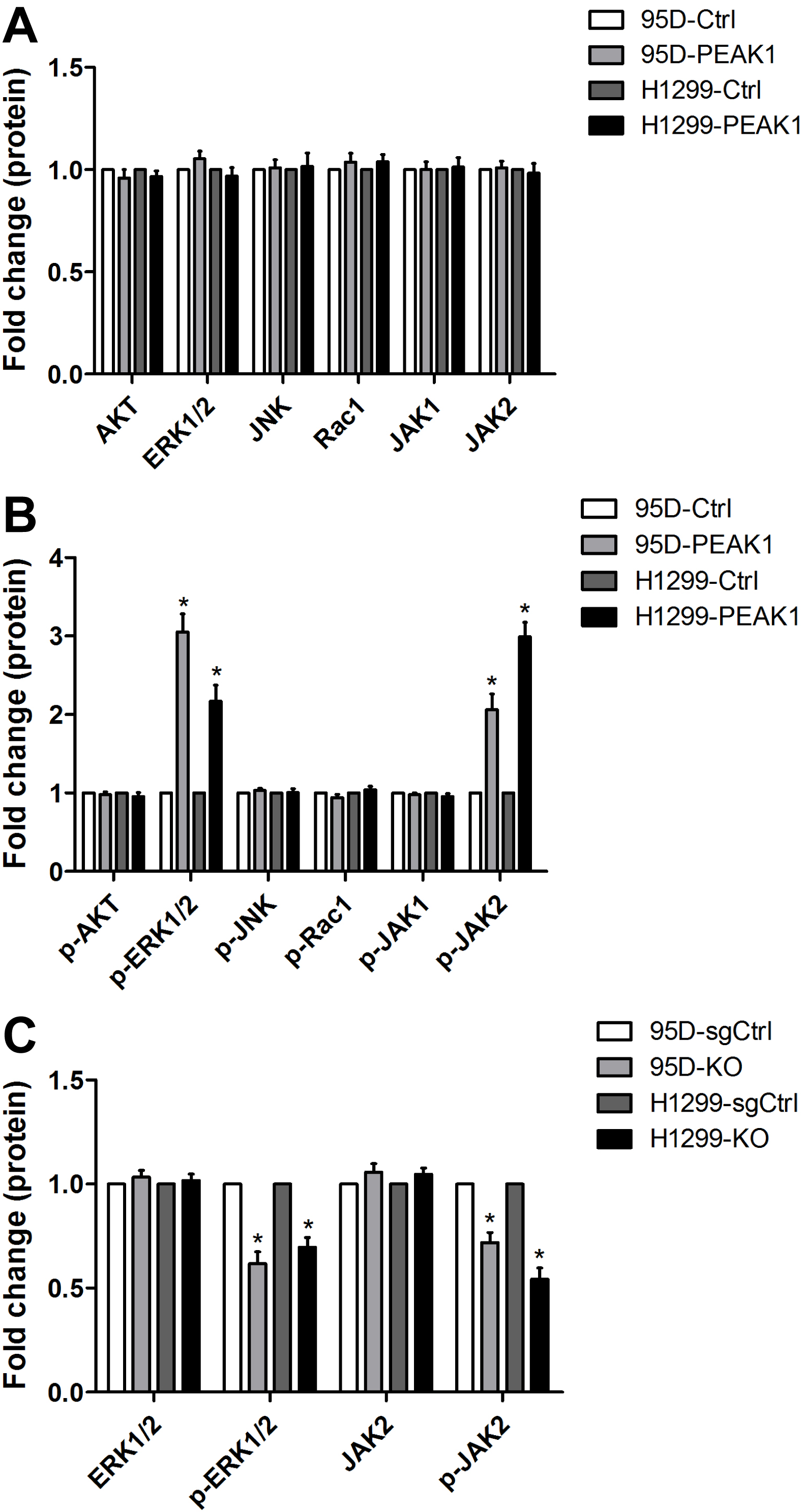

Supplement: Supplementary file 5 — Figure S5 [file 41419_2018_817_MOESM5_ESM.tif]

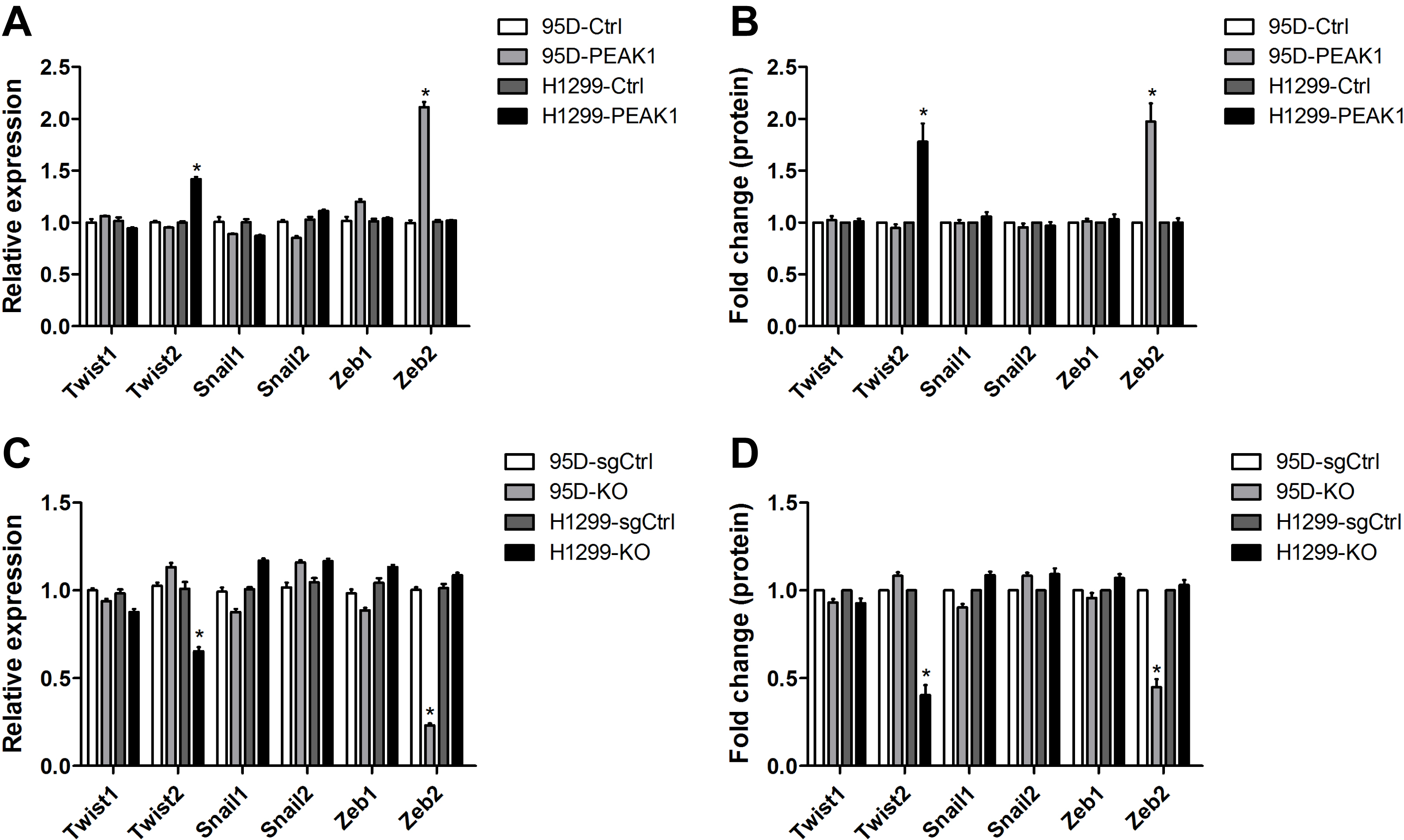

Supplement: Supplementary file 6 — Figure S6 [file 41419_2018_817_MOESM6_ESM.tif]

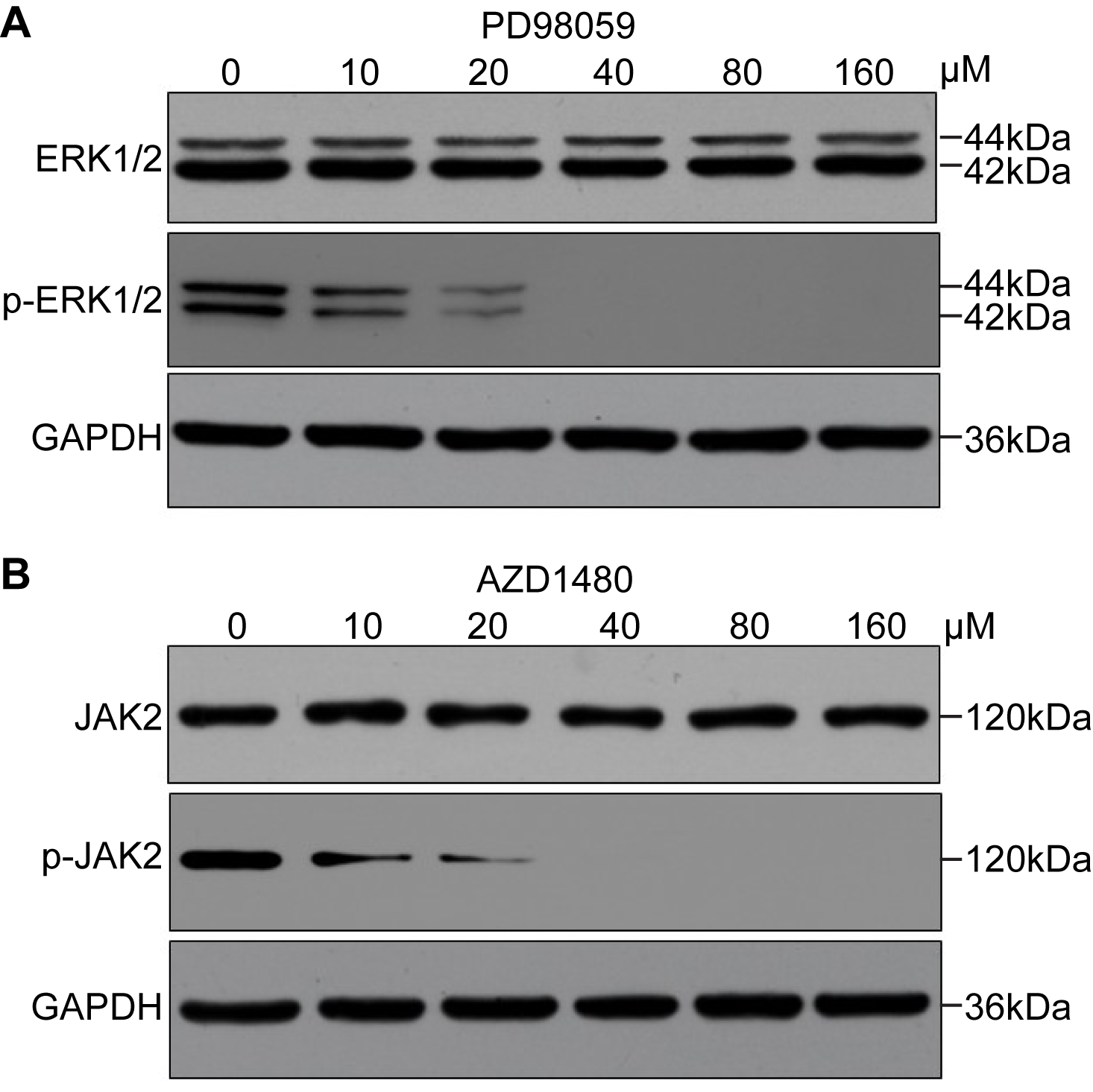

Supplement: Supplementary file 7 — Figure S7 [file 41419_2018_817_MOESM7_ESM.tif]

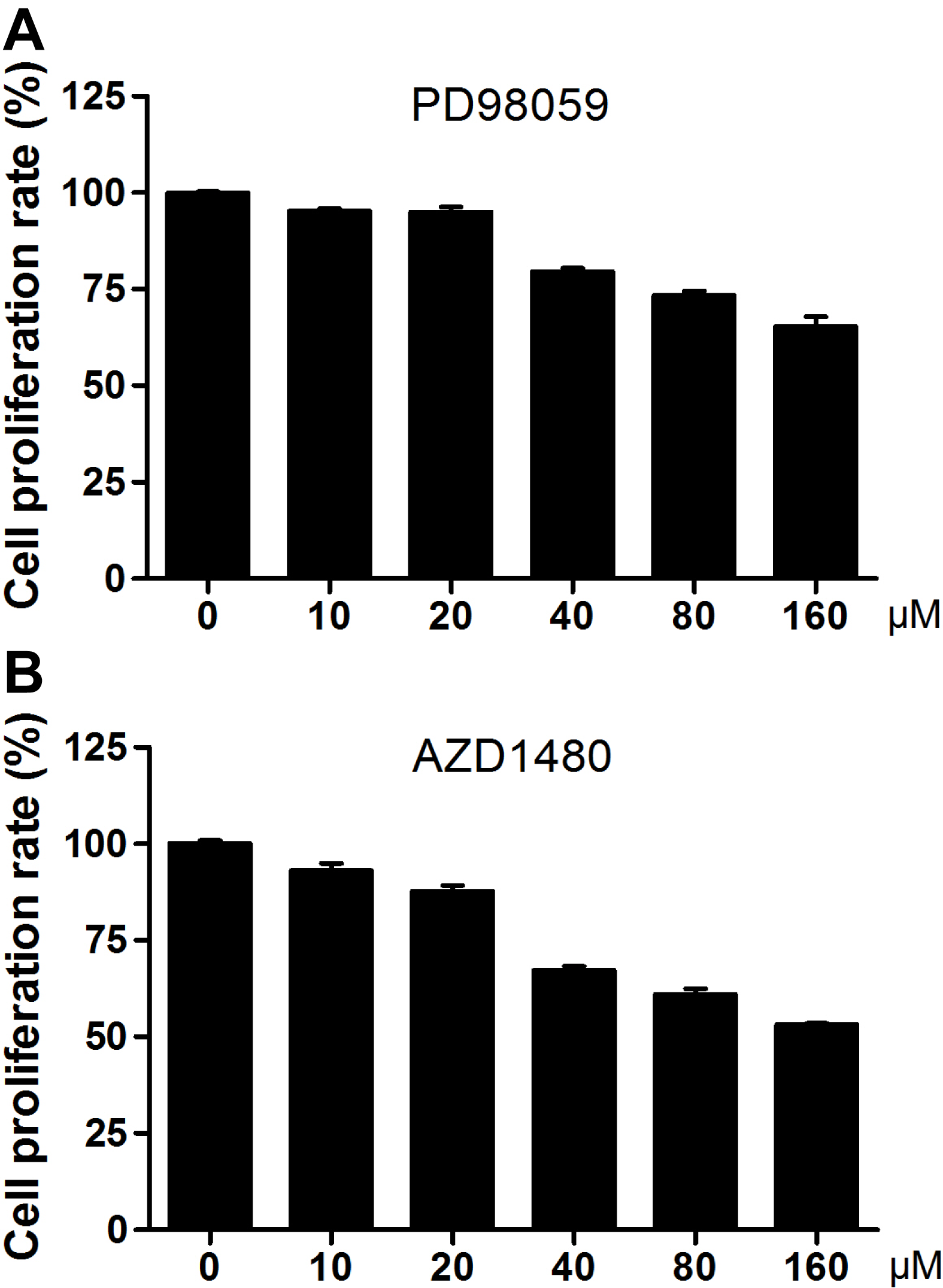

Supplement: Supplementary file 8 — Figure S8 [file 41419_2018_817_MOESM8_ESM.tif]

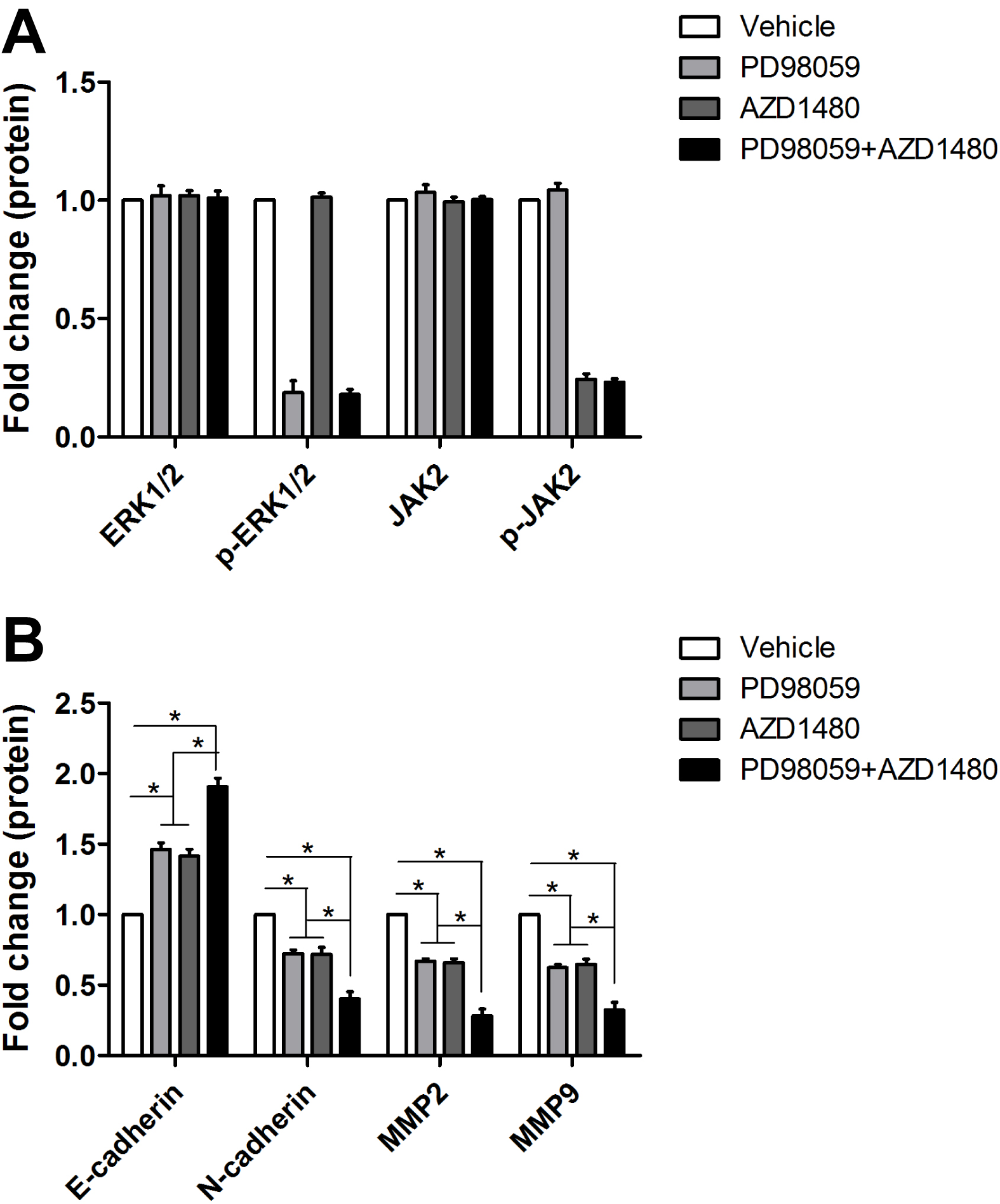

Supplement: Supplementary file 9 — Figure S9 [file 41419_2018_817_MOESM9_ESM.tif]
