# Supplementary material for: Overexpression of PEAK1 contributes to epithelial–mesenchymal transition and tumor metastasis in lung cancer through modulating ERK1/2 and JAK2 signaling
Source: Cell Death Dis. 2018 Jul 23;9(8):802. doi: 10.1038/s41419-018-0817-1 (PMC6056550; doi:10.1038/s41419-018-0817-1)
Supplement: Supplementary file 10 — Table S1 [file 41419_2018_817_MOESM10_ESM.docx]

| Groups | Cases | PEAK1 expression | | | *P*-value |
| --- | --- | --- | --- | --- | --- |
|  |  | Negative | Positive | Positive rate (%) |  |
| Carcinoma tissues | 70 | 26 | 44 | 62.86 | ＜0.001 |
| Normal tissues | 34 | 27 | 7 | 20.59 |  |
