# Supplementary material for: Overexpression of PEAK1 contributes to epithelial–mesenchymal transition and tumor metastasis in lung cancer through modulating ERK1/2 and JAK2 signaling
Source: Cell Death Dis. 2018 Jul 23;9(8):802. doi: 10.1038/s41419-018-0817-1 (PMC6056550; doi:10.1038/s41419-018-0817-1)
Supplement: Supplementary file 11 — Supplementary Figure and Table Legends [file 41419_2018_817_MOESM11_ESM.docx]

**Supplementary Figure and Table Legends**

**Fig. S1 PEAK1 expression does not affect the NSCLC cell proliferation.** **a** The proliferation ability of 95D-Ctrl and 95D-PEAK1 cells was detected by CCK-8 assay. b PEAK1 overexpression cannot affect the proliferation ability of H1299 cells. **c** and **d** The proliferation of 95D-sgCtrl, 95D-KO, H1299-sgCtrl and H1299-KO cells was assessed (two clones, **a** and **b**). Columns are the average of three independent experiments ± SEM.

**Fig. S2 TGF-β-EMT process does not affect the levels of PEAK1 in NSCLC cells. a** The mRNA levels of E-cadherin, N-cadherin and PEAK1 in 95D cells with or without TGF-β (10 ng/ml) treatment for 48 hours was detected by qRT-PCR. **b** qRT-PCR assay of the mRNA expression of E-cadherin, N-cadherin and PEAK1 in H1299 cells with or without TGF-β(10 ng/ml) treatment for 48 hours. **c** Western blot analysis of the expression of E-cadherin, N-cadherin and PEAK1 in 95D and H1299 cells with or without TGF-β (10 ng/ml) treatment for 48 hours. **d** The levels of E-cadherin, N-cadherin and PEAK1 protein were calculated. **P*<0.05

**Fig. S3 PEAK1 overexpression does not upregulate the expression of TGF-β** **in NSCLC cells. a** The mRNA expression of TGF-β1/2/3 in 95D-Ctrl and 95D-PEAK1 cells was detected by qRT-PCR. **b** qRT-PCR assay of the mRNA expression of TGF-β1/2/3 in H1299-Ctrl and H1299-PEAK1 cells.

**Fig. S4 PEAK1 expression promotes the expression of N-cadherin mRNA, but reduces the expression of E-cadherin mRNA in NSCLC cells.** a The mRNA expression of E-cadherin and N-cadherin in 95D-Ctrl, 95D-PEAK1, H1299-Ctrl and H1299-PEAK1 cells was detected by qRT-PCR. **b** qRT-PCR assay of the mRNA expression of E-cadherin and N-cadherin in 95D-sgCtrl, 95D-KO, H1299-sgCtrl and H1299-KO cells. **P*<0.05

**Fig. S5 PEAK1 expression promotes ERK1/2 and JAK2 signaling in NSCLC cells.** **a** The levels of AKT, ERK1/2, JNK, Rac1, JAK1 and JAK2 in 95D-Ctrl, 95D-PEAK1, H1299-Ctrl and H1299-PEAK1 cells were calculated. b The levels of p-AKT, p-ERK1/2, p-JNK, p-Rac1, p-JAK1 and p-JAK2 in 95D-Ctrl, 95D-PEAK1, H1299-Ctrl and H1299-PEAK1 cells were calculated. **c** The levels of ERK1/2, p-ERK1/2, JAK2 and p-JAK2 in 95D-sgCtrl, 95D-KO, H1299-sgCtrl and H1299-KO cells were calculated. The data are representative of at least three different experiments ± SEM. **P*<0.05

**Fig. S6 PEAK1 expression enhances Zeb2 or Twist2 expression in NSCLC cells.** **a** and **b** qRT-PCR and western blot showed upregulated expression of Zeb2 in 95D-PEAK1 cells, and upregulated expression of Twist2 in H1299-PEAK1 cells compared with the corresponding control cells. **c** and **d** The expression of TFs in 95D-sgCtrl, 95D-KO, H1299-sgCtrl and H1299-KO cells detected by qRT-PCR and western blot assays (two clones, **a** and **b**). The data are representative of at least three different experiments ± SEM. **P*<0.05

**Fig. S7 PD98059 or AZD1480 prohibits the activation of ERK1/2 or JAK2 signaling in a concentration dependent manner.** **a** Effect of different PD98059 concentration on the activation of ERK1/2 in 95D-PEAK1 cells. **b** Effect of different AZD1480 concentration on the activation of JAK2 in 95D-PEAK1 cells.

**Fig. S8 Effect of different PD98059 or AZD1480 concentration on the proliferation of 95D-PEAK1 cells in vitro.** **a** The proliferation rate of 95D-PEAK1 cells was analyzed by CCK-8 assay in different PD98059 concentration. **b** The proliferation rate of 95D-PEAK1 cells was analyzed by CCK-8 assay in different AZD1480 concentration.

**Fig. S9 Pharmacological inhibitors of ERK1/2 and JAK2 reduce PEAK1-induced EMT. a** The levels of ERK1/2, p-ERK1/2, JAK2 and p-JAK2 of 95D-PEAK1 cells in the absence (vehicle), or presence of 20 μM PD98059 and/or AZD1480 were calculated. **b** The levels of E-cadherin, N-cadherin, MMP2 and MMP9 of 95D-PEAK1 cells in the absence (vehicle), or presence of 20 μM PD98059 and/or AZD1480 were calculated. The data are representative of at least three different experiments ± SEM. **P*<0.05

**Table S1 Upregulation of PEAK1 in lung cancer tissues**
